# Temporal summation of mechanical pain prospectively predicts movement-evoked pain severity in adults with chronic low back pain

**DOI:** 10.1186/s12891-021-04306-5

**Published:** 2021-05-10

**Authors:** Demario S. Overstreet, Ava N. Michl, Terence M. Penn, Deanna D. Rumble, Edwin N. Aroke, Andrew M. Sims, Annabel L. King, Fariha N. Hasan, Tammie L. Quinn, D. Leann Long, Robert E. Sorge, Burel R. Goodin

**Affiliations:** 1 Department of Psychology, University of Alabama at Birmingham, 1300 University Boulevard, Campbell Hall, Suite 237, Birmingham, AL 35294 USA; 2 School of Nursing, Nurse Anesthesia Program, Department of Acute, Chronic, & Continuing Care, University of Alabama at Birmingham, Birmingham, USA; 3 School of Public Health, Department of Biostatistics, University of Alabama at Birmingham, Birmingham, USA

**Keywords:** Low back pain, Movement, Temporal summation, Conditioned pain modulation

## Abstract

**Background:**

Biopsychosocial factors above and beyond pathoanatomical changes likely contribute to the severity of chronic low back pain. A pro-nociceptive endogenous pain modulatory balance (↓inhibition and ↑facilitation) may be an important contributor to chronic low back pain severity and physical function; however, additional research is needed to address this possibility. The objective of this study was to determine whether quantitative sensory tests of endogenous pain inhibition and facilitation prospectively predict movement-evoked pain and cLBP severity self-reported on a validated questionnaire.

**Methods:**

One hundred thirty-four individuals with chronic low back pain were enrolled in this two-session study. During the first study session, temporal summation of mechanical pain and conditioned pain modulation were assessed at the lumbar spine to determine endogenous pain facilitation and inhibition, respectively. One week later, participants returned for a second study session whereby they reported their pain severity and pain interference using the Brief Pain Inventory-Short Form. Movement-evoked pain and physical function capacity were assessed upon completion of the balance, walking, and transition from sit to stand tests of the Short Physical Performance Battery.

**Results:**

Temporal summation of mechanical pain, but not conditioned pain modulation, significantly and prospectively predicted greater movement-evoked pain and poorer physical function on the Short Physical Performance Battery. Neither temporal summation nor conditioned pain modulation were significantly related to self-reported pain severity or pain interference on the Brief Pain Inventory-Short Form.

**Conclusions:**

Findings suggest that a pro-nociceptive pain modulatory balance characterized by enhanced pain facilitation may be an important driver of movement-evoked pain severity and poor physical function in individuals with chronic low back pain.

## Introduction

Low back pain is one of the most common disabling conditions in the world [1]. The worldwide point prevalence of activity-limiting (acute and chronic) low back pain is approximately 12% [2], which equates to approximately 933 million people globally suffering with low back pain at any given time. Chronic low back pain (cLBP) refers to pain lasting at least 12 week or longer, and it is consistently among the top five most common reasons for primary care physician visits [3]. This is particularly alarming given the substantial (and growing) direct and indirect economic costs associated with cLBP [3]. In line with growing prevalence and expense, the last two decades saw steadily increased utilization of interventions targeting cLBP, including surgical, pharmacological, and non-pharmacological approaches [4–6]. Yet despite increased utilization, sustained pain relief and functional restoration is rarely achieved for those with cLBP. The vast majority of cLBP is “non-specific” and is not accompanied by readily identifiable pathology of the spine or related tissues [7, 8]. Without a clear target for treatment of cLBP, effective pain management can be difficult to achieve [9].

Even when pathoanatomical changes in the spine are detected, there is often poor correspondence between these diagnostic measures of cLBP and clinical symptoms [10, 11] This suggests that factors above and beyond pathoanatomy must contribute to cLBP severity. In recent years, a growing number of case-control studies have revealed that individuals with cLBP demonstrate greater dysfunction in endogenous pain modulatory pathways compared to controls using experimental pain protocols (i.e., quantitative sensory testing or QST) [12–14]. Similarly, a cross-sectional study addressing this topic found that augmented pain sensitivity and dysfunctional endogenous pain modulation were associated with greater cLBP severity and disability [15]. Emerging evidence suggests that cLBP severity is related to a pro-nociceptive pain modulatory balance [16]; however, much of this evidence has been cross-sectional, making it difficult to ascertain the directionality of the relationships. Whether QST-based tests of endogenous pain modulatory balance might be useful for prospectively predicting future reports of cLBP severity has received less attention.

Dynamic forms of QST that include tests of temporal summation (TS) of pain and conditioned pain modulation (CPM) are likely best suited to address this question given the growing evidence base attesting to the clinical relevance of each [17]. TS of pain is a QST method that invokes neural mechanisms related to pain facilitation [18], while CPM invokes neural mechanisms related to pain inhibition [19]. Taken together, TS and CPM measures are thought to induce a process of modulation believed to reflect the “real-life” endogenous modulation exerted by patients when exposed to clinical pain [20]. Typically, patients with clinical pain of various types express either less efficient CPM or enhanced TS, or both [17, 20].

The vast majority of past research examining CPM and TS in relation to cLBP severity has incorporated validated self-report questionnaires of pain recall as the clinically-relevant index of pain severity [12–15]. Findings have been mixed with some studies reporting significant associations between CPM and/or TS and pain recall [12, 15], while others reported no such significant associations [13]. From this body of research, it would appear that the utility of QST measures for predicting cLBP severity is limited. However, it should be considered that pain severity recalled on self-report questionnaires does not fully capture the complexity of cLBP. Individuals with musculoskeletal pain conditions including cLBP often experience significant movement-evoked pain upon completion of physical activity [21]. Emerging evidence has revealed distinct differences between pain recalled on self-report questionnaires and movement-evoked pain [22]. A key mechanism of movement-evoked pain is the activation of silent nociceptors in response to joint movement or other movement-related stimuli that are not normally painful [21, 23]. Therefore, movement-evoked pain represents a distinct pain-related phenomenon not generally reflected by pain recall, and it may be a particularly important measure of cLBP severity above and beyond traditional self-report questionnaires. Whether TS of pain and CPM might differentially predict severity of movement-evoked pain versus pain recalled on a validated questionnaire is a topic that remains to be adequately addressed.

This study included a community-dwelling sample of adults with non-specific cLBP. The objective was to determine whether TS of pain and/or CPM prospectively predicted movement-evoked pain severity and pain reported on a validated questionnaire, each assessed 1-week later. It was hypothesized that, controlling for demographic and clinical covariates, enhanced TS of mechanical pain and diminished CPM would each prospectively predict greater movement-evoked pain severity and severity of pain recalled on a validated questionnaire. A secondary hypothesis was that TS of mechanical pain and CPM would similarly predict poorer physical function on the SPPB and greater pain interference on the validated questionnaire.

## Methods

### Study overview

**Fig. 1:**
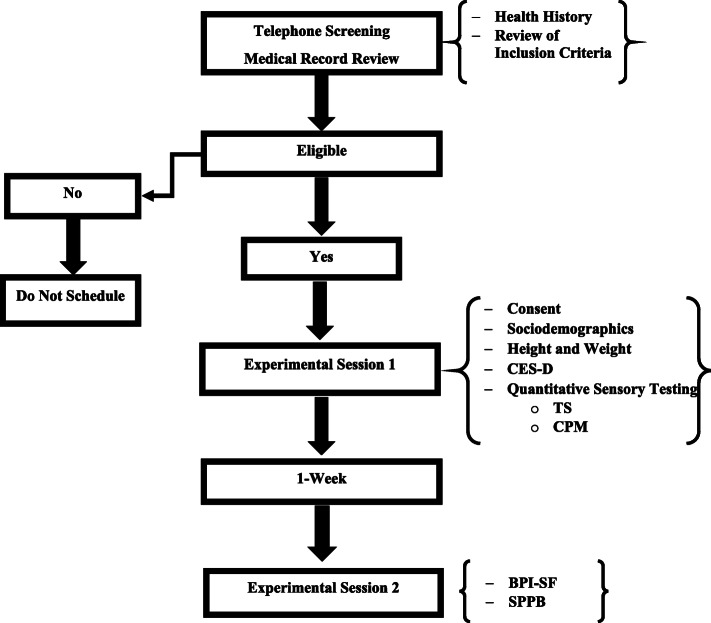
Flow Diagram

This study was part of an ongoing parent project investigating ethnic/racial and socioeconomic differences in cLBP severity and disability (Examining Racial And SocioEconomic Disparities in cLBP; ERASED). The parent project employs a biopsychosocial conceptual rubric that examines biobehavioral, psychological, and sociocultural factors that may help explain differences in cLBP between non-Hispanic Black and non-Hispanic White adults. Participant data presented in this study were collected between November 2017 and January 2020. The procedures and experimental methods described below are limited to those involved in the current research study. A flow diagram illustrating matriculation through the current study is presented in Fig. 1. Interested participants completed telephone-based screening to determine initial study eligibility; health history was also reviewed via electronic medical records. Eligible participants completed two distinct laboratory-based study sessions separated by 1 week. Participants completed a comprehensive QST battery during the first study session. TS of pain was examined via mechanical stimuli, whereas CPM was examined with algometry (test stimulus) and the cold pressor task (conditioning stimulus). Approximately 1 week later, participants returned to the laboratory to complete their second study session. The session included assessments of movement-evoked pain and physical function using the standardized Short Physical Performance Battery (SPPB) [24]. Movement-evoked pain was assessed upon completion of each task (balance, walking, and transition from sit to stand) that comprises the SPPB. Participants also completed the Brief Pain Inventory – Short Form (BPI-SF, described in greater detail below), which is a validated questionnaire of self-reported pain severity and interference [25]. Potentially confounding variables were also measured including both the demographic (e.g., sex, race, age, socioeconomic status) and clinical (e.g., body mass index, current opioid prescription, depressive symptoms) characteristics of this sample with cLBP. This study was conducted in accordance with the cLBP research standards put forth by the Research Task Force of the National Institutes of Health Pain Consortium [26]. All procedures were reviewed and approved by the University of Alabama at Birmingham (UAB) Institutional Review Board, and carried out in a manner consistent with ethical research guidelines as outlined in the Declaration of Helsinki.

### Participants

Community-dwelling participants with cLBP were recruited via flyers posted at the UAB Pain Treatment Clinic and surrounding community. Individuals were included in the study if low back pain had reportedly persisted for at least three consecutive months and was present for at least half the days in the past 6 months [27]. The primary pain complaint had to be low back pain with non-specific origin. In order to examine the full range of cLBP severity, there was no minimum threshold of self-reported pain intensity for inclusion in this study. A total of 138 participants with cLBP were enrolled and included in this study; however, four participants did not provide complete study data and were excluded from the present analysis. This resulted in a final sample size of 134 participants with cLBP. All participants provided informed consent prior to commencing study involvement.

### Procedures

#### Telephone screening and medical record review

A telephone-based screening was completed for each participant to review study inclusion and exclusion criteria. This was followed by review of electronic medical records to confirm cLBP status and that all inclusion criteria were met. Electronic medical records also provided information pertaining to other health comorbidities and current prescriptions for analgesic medications. A comprehensive list of inclusion and exclusion criteria for the parent project from which this study’s participants were sampled has previously been reported [28]. All participants had to be between the ages of 18 and 85 years; able to read, write, and understand English; and self-identify as non-Hispanic Black/African American or non-Hispanic White/Caucasian. Participants were excluded from study participation if their low back pain was attributable to other factors such as ankylosing spondylitis, infection, malignancy, or compression fracture. Further, there must not have been any evidence of surgical intervention or accident/trauma within the past 12 months. Excluding those with a surgery or accident/trauma in the past 12 months mitigated the potential impact of any acute injury on reported cLBP severity. Additional exclusion criteria included: 1) Presence of systemic rheumatic conditions (e.g. rheumatoid arthritis, systemic lupus erythematosus, fibromyalgia); 2) Evidence of uncontrolled hypertension (i.e. SBP/DBP > 150/95), cardiovascular or peripheral arterial disease; 3) Poorly controlled diabetes (HbA1c > 7%); 4) Neurological disease (e.g. Parkinson’s, multiple sclerosis, epilepsy); 5) Serious psychiatric disorder requiring hospitalization within the past 12 months; and 6) Pregnancy. Given that participants were primarily recruited from a pain treatment clinic, many were actively prescribed daily analgesic medications including opioids. Participants using daily opioids were not excluded as this could have undermined the generalizability of study results. Furthermore, they were not asked to withhold opioid pain medications on the days of study participation. This is because temporary withdrawal from these medications could have affected pain perception. Rather, opioid medications currently prescribed for pain were recorded and controlled in statistical analyses as needed.

#### Experimental study session 1

Participants initially provided sociodemographic information that included race/ethnicity, age, sex/gender, and annual household income. Height and weight were collected for calculation of body mass index (BMI) prior to completion of a standardized depressive symptoms measure. Next, participants were asked to lay prone on a medical grade massage table in order to complete a QST battery designed to assess endogenous pain modulatory balance. The QST battery specifically included controlled sensory stimulation procedures to assess endogenous pain facilitatory processes - TS of mechanical pain, as well as endogenous pain inhibitory processes - CPM. For this study, TS of mechanical pain was examined exclusively as a measure of endogenous pain facilitation. Previous research has demonstrated that TS of mechanical pain is more clinically relevant than TS of thermal pain for predicting musculoskeletal clinical pain severity [15, 29, 30].

##### Center for Epidemiological Studies-Depression Scale (CES-D)

Depressive symptoms were assessed using the CES-D [31]. This 20-item measure assesses the frequency of experiencing depressive symptoms over the past week (0 – never or rarely to 3 – most of the time/all the time). Symptoms of depression measured by the CES-D include negative mood, guilt/worthlessness, helplessness/hopelessness, psychomotor retardation, loss of appetite, and sleep disturbance. This measure has been shown to be reliable and valid in general populations, including when used in chronic pain populations. Responses are summed (range 0–60), with higher scores indicating greater severity of depression. The CES-D in this study demonstrated excellent internal consistency (Cronbach’s α = .915).

##### Temporal summation of mechanical stimulation

TS of mechanical stimulation was assessed at the erector spinae muscles of the lumbar spine using a weighted (512 mN) pinprick stimulator (MRC Systems, Heidelberg, Germany) [32]. The pinprick stimulator was oriented perpendicularly and held just above the intended point of contact. The punctate probe was then lowered gently until the fine weighted probe retracted fully inside of the probe’s hollow metal cylinder, creating the desired standardized stimulation. Participants were first subjected to a single contact from the pinprick stimulator and prompted to rate the pain intensity resulting from this sensation using a 0–100 numeric rating scale, where *“0 = no pain and 100 = most intense pain imaginable”*. Next, the pinprick stimulator was applied 10 successive times at a rate of one contact per second. Participants were again asked to provide a single 0–100 rating indicating the greatest intensity of pain experienced during the 10 repeated contacts. This procedure was repeated twice at the lumbar spine. Pain ratings for the single and multiple contacts performed at each anatomical location were averaged across the two trials. TS effects (i.e., Δ change score) at the lumbar spine were calculated by subtracting the pain intensity ratings following the first contact from the ratings following the series of 10 contacts.

##### Conditioned pain modulation

CPM was tested at the erector spinae muscles of the lumbar spine using algometry as the test stimulus and hand immersion into the cold pressor as the conditioning stimulus [19]. A handheld algometer (Medoc, Ltd., AlgoMed, Ramat Yishai, Israel) was applied three times at the lumbar region to determine participants’ baseline pressure pain thresholds (PPTs). Pressure was gradually increased at a rate of 30 kilopascals (kPa) per seconds, and participants indicated when the increasing pressure stimulation first became painful. PPTs were measured in kilopascals (kPa). Following baseline PPT determination, participants underwent a series of two cold pressor immersions that consisted of placing the left hand, up to the wrist, into 12 °C circulating cold water for 1 min. The cold pressor was maintained at 12 °C by an ARTIC A25 refrigerated bath with an SC150 immersion circulator (ThermoFisher Scientific, USA) that constantly circulated the water to prevent local warming around the submerged hand. Our previous work has indicated this temperature to be best for maximizing a full 1 min hand immersion, while also producing a moderate amount of pain (~ 50 ± 10 on the 0–100 numeric rating scale) [33]. Immediately upon removal of the hand from the cold pressor, the algometer was again used to deliver noxious mechanical stimulation to the lumbar region. Participants again indicated when the increasing pressure stimulation first became painful, which represented their conditioned PPTs. There was a 2-min rest period between each CPM trial. The three baseline PPTs were averaged as were the two conditioned PPTs from the CPM trials. CPM effects were calculated as a percent change from baseline according to the following formula:

$$ \left(\left(\mathrm{Conditioned}\ \mathrm{PPT}\hbox{-} \mathrm{Baseline}\;\mathrm{PPT}\right)/\mathrm{Baseline}\ \mathrm{PPT}\right)\ast 100 $$

#### Experimental study session 2

Approximately 1-week after completing the first experimental session, each participant returned to the laboratory and engaged in a second experimental study session. This included completion of the BPI-SF pain questionnaire, as well as assessment of movement-evoked pain and physical function.

##### Brief pain inventory - short form

The BPI-SF is a multidimensional pain scale used to assess self-reported pain severity and its interference with daily functioning [25]. The questionnaire is composed of four items asking about pain severity (worst pain, least pain, average pain, and pain right now) over the past 24 h. Additionally, seven items assess the degree to which pain interferes with functioning in the following domains: general activity, mood, walking ability, normal work, relations with other people, sleep, and enjoyment of life. Each item is scored from 0 (no pain or does not interfere) to 10 (worst imaginable pain or completely interferes). Higher scores suggest greater pain severity and pain interference. The BPI-SF is a well validated chronic pain questionnaire that has previously been used in samples with cLBP [34]. The BPI-SF in this study demonstrated excellent internal consistency (Cronbach’s α = .951).

##### Short physical performance battery

The SPPB assesses lower extremity function with three movement tasks: standing balance, 4-m walking speed, and ability to rise from a chair [24]. Specifically, participants completed the following movement tasks in consecutive order: 1) Stand with their feet oriented in the side-by-side, semi-tandem, and tandem positions for 10 s each; 2) Rise from, and return to, a seated position in a chair five times; and 3) Walk a distance of four-meters, twice. For each movement, they received a score of 0–4 (total score 0–12) based on their performance. If participants did not feel safe completing any of the SPPB tasks, they were given a score of zero to denote *non-*participation. A lower score on the SPPB is indicative of worse physical function, and greater likelihood of disability. After completion of each movement task, participants were asked to provide a pain intensity rating for any movement-evoked pain experienced during completion of the balance, chair, and walking tests. The 0–100 numeric rating scale was again utilized for this purpose, whereby: (*0 = no pain and 100 = most intense pain imaginable).* The SPPB is standardized and has been well validated for use in populations with cLBP [35, 36].

#### Data analysis

All data were analyzed using SPSS, version 25 (IBM; Armonk, NY). Descriptive statistics were computed and represented as percentages or means (standard deviations). Group differences among potential covariates of interest (e.g., race/ethnicity and sex/gender) were examined using independent samples t-tests. Paired t-tests were used to examine differences within individuals between 1 and 10 contacts for TS of mechanical pain and between baseline and conditioned PPTs for CPM. The strength and direction of associations among continuous variables were examined using Pearson’s correlations. Sequential hierarchical multiple regression models were employed to investigate the extent to which experimentally-induced TS of mechanical pain and CPM prospectively predicted pain and physical function outcomes, controlling for demographic and clinical characteristics. Demographic characteristics were entered in *step 1* of the hierarchical regression models, while clinical characteristics were entered in *step 2,* followed by TS of mechanical pain and CPM in *step 3*. The level of statistical significance was 0.05.

## Results

### Participant characteristics

Descriptive characteristics for the sample of participants living with cLBP are shown in Table 1*.* The average age of the sample was 45.4 (SD = 14.1) and ranged from 18 to 82 years. This sample was comprised of more female (56.7%) than male participants (43.3%). Further, 61.2% of the sample self-identified as Non-Hispanic Black or African American; the remaining participants indicated their race/ethnicity to be Non-Hispanic White or Caucasian (38.8%). The largest portion of the sample (33.8%) reported their annual household income to be between $0 and $19,999. The average BMI across all participants was 31.1 (SD = 7.4). The mean score for depressive symptoms was 17.6 (SD = 10.5) as indicated by CES-D; scores on the CES-D ranged from 0 to 47. Medical record review confirmed that 39.6% of the sample had a current and active prescription for an opioid analgesic. The average rating of movement-evoked pain on the SPPB was 26.4 (SD = 27.4); observed scores ranged from 0 (no pain) to 100 (most intense pain imaginable). Furthermore, physical function scores on the SPPB ranged from 3 to 12 (lower scores are suggestive of greater disability); the sample mean for SPPB physical function was 9.5 (SD = 1.9). Analysis of Q-Q plots and the Shapiro-Wilk tests (*p* < .05) revealed that the residuals of movement-evoked pain and physical function on the SPPB were not normally distributed. Lastly, mean self-reported pain severity on the BPI-SF was 4.7 (SD = 2.4) and ranged from 0 to 9.5, while mean self-reported pain interference on the BPI-SF was 3.7 (SD = 2.6) and ranged from 0 to 9.3.

**Table 1:** Descriptive characteristics and clinical information for cLBP participants (*N* = 134)

**Demographic characteristics**	**Mean (SD) or %**	**Range**
Age (years)	45.4 (14.1)	18 to 82
Sex (% female)	56.7%	
Race (% African American)	61.2%	
Annual household income		
$0–19,999	33.8%	
$20,000 – 34,999	12.5%	
$35,000 – 49,999	13.2%	
$50,000 – 74,999	15.4%	
$75,000 – 99,999	7.4%	
100,000 and Greater	14.0%	
**Clinical characteristics**	**Mean (SD) or %**	
Current opioid prescription (% yes)	39.6%	
BMI (weight/height^2^)	31.1 (7.4)	19.1 to 64.6
Depressive symptoms (CES-D)	17.6 (10.5)	0 to 47
Movement evoked pain (SPPB)	26.4 (27.4)	0 to 100
Physical function (SBBP)	9.5 (1.9)	3 to 12
Pain severity (BPI-SF)	4.7 (2.4)	0 to 9.5
Pain interference (BPI-SF)	3.7 (2.6)	0 to 9.3

*BMI* body mass index, *CES-D* Center for Epidemiological Studies – Depression Scale, *SPPB* Short Physical Performance Battery, *BPI-SF* Brief Pain Inventory – Short Form

### TS and CPM effects

Comparative results for TS of mechanical pain and CPM are presented in Table 2. For TS of mechanical pain (512mN), the pain intensity rating elicited by the first contact was compared to the pain intensity rating elicited following 10 successive contacts. A paired t-test revealed that mean pain intensity ratings following 10 successive contacts were significantly greater than mean ratings for the first contact (t = 10.64, *p* < .001). This statistically significant TS effect is suggestive of endogenous pain facilitation at the site of the erector spinae muscles of the lumbar spine. For analysis of CPM effects, mean baseline PPT was compared to mean conditioned PPT. A paired t-test did not reveal statistically significant evidence of a CPM effect at the erector spinae muscles of the lumbar spine (t = 1.94, *p* = .054). This non-statistically significant CPM effect is likely indicative of diminished endogenous pain inhibitory capacity at the lumbar spine. Analysis of Q-Q plots and the Shapiro-Wilk tests (*p* < .05) revealed that the residuals of TS of mechanical pain and CPM were not normally distributed.

**Table 2:** Temporal summation (TS) and conditioned pain modulation (CPM) effects

	Mean (SD)	Sig.
**TS of mechanical pain (512mn) at the low back**		
1 Contact	32.6 (31.7)	
10 Contacts	50.1 (33.7)	t = 10.54, *p* < .001
**CPM at the low back**		
Baseline PPT	411.6 (213.2)	
Conditioned PPT	430.6 (221.4)	t = 1.94, *p* = .054

Note: 1 Contact = pain intensity rating (0–100) in response to first contact with mechanical stimuli, 10 contact = pain intensity rating (0–100) in response to 10 contacts with mechanical stimuli; *PPT* pressure pain threshold measured in kilopascals (kPa)

### Zero-order correlations

Results from the correlation analysis are displayed in Table 3. TS of mechanical pain was significantly linearly associated with greater movement-evoked pain (*r* = .206, *p* = .017) and poorer physical function (*r* = −.201, *p* = .021) on the SPPB. Conversely, TS of mechanical pain was not significantly associated with self-reported pain severity (*r* = .096, *p* = .271) or pain interference (*r* = .121, *p* = .163) on the BPI-SF. CPM was not significantly associated with pain severity or physical function/interference on either the SPPB or BPI-SF (all p’s > .05). Movement-evoked pain and physical function on the SPPB were each significantly associated with self-reported pain severity and pain interference on the BPI-SF. Greater CPM was significantly correlated with greater TS of mechanical pain (*r* = .228, *p* = .008).

**Table 3:** Pearson correlations

Variable	1	2	3	4	5
1. TS mechanical – 512mN	___				
2. CPM	.228**	___			
3. Movement evoked pain (SPPB)	.206*	.051	___		
4. Physical function (SPPB)	−.201*	−.038	−.527**	___	
5. Pain severity (BPI-SF)	.096	−.035	.726**	−.484**	___
6. Pain interference (BPI-SF)	.121	−.028	.667**	−.569**	.790**

*TS* temporal summation, *mN* milliNewton, *CPM* conditioned pain modulation, *SPPB* Short Physical Performance Battery, *BPI-SF* Brief Pain Inventory – Short Form

* = *p* < .05, ** = *p* < .01

### Covariates of interest

Male participants reported significantly greater movement-evoked pain than female participants (t = 2.74, *p* = .007). Compared to their non-Hispanic White counterparts, non-Hispanic Black participants had significantly greater movement-evoked pain (t = 2.70, *p* = .008) and poorer physical function (t = 2.20, *p* = .030) on the SPPB, as well as greater self-reported pain severity (t = 3.33, *p* = .001) and interference (t = 2.57, *p* = .011) on the BPI-SF. Increasing age was significantly associated with greater movement-evoked pain (*r* = .185, *p* = .033) and poorer physical function (*r* = −.309, *p* < .001) on the SPPB. Lower annual household income was significantly associated with greater pain severity and poorer physical function on the SPPB and BPI-SF (all p’s < .001). Current opioid prescription and BMI were not significantly associated with any of the pain severity or physical function scales of the SPPB or BPI-SF. However, greater depressive symptoms was significantly associated with greater movement-evoked pain (*r* = .262, *p* = .002) and poorer physical function (*r* = −.240, *p* = .005) on the SPPB, as well as greater self-reported pain severity (*r* = .401, *p* < .001) and interference (*r* = .498, *p* < .001) on the BPI-SF. Given their theoretical and empirical relevance, participant sex, annual household income, age, race, BMI, current opioid prescription, and depressive symptoms were all included as statistical covariates in the hierarchical regression models presented below.

### Hierarchical multiple regression models

**Fig. 2:**
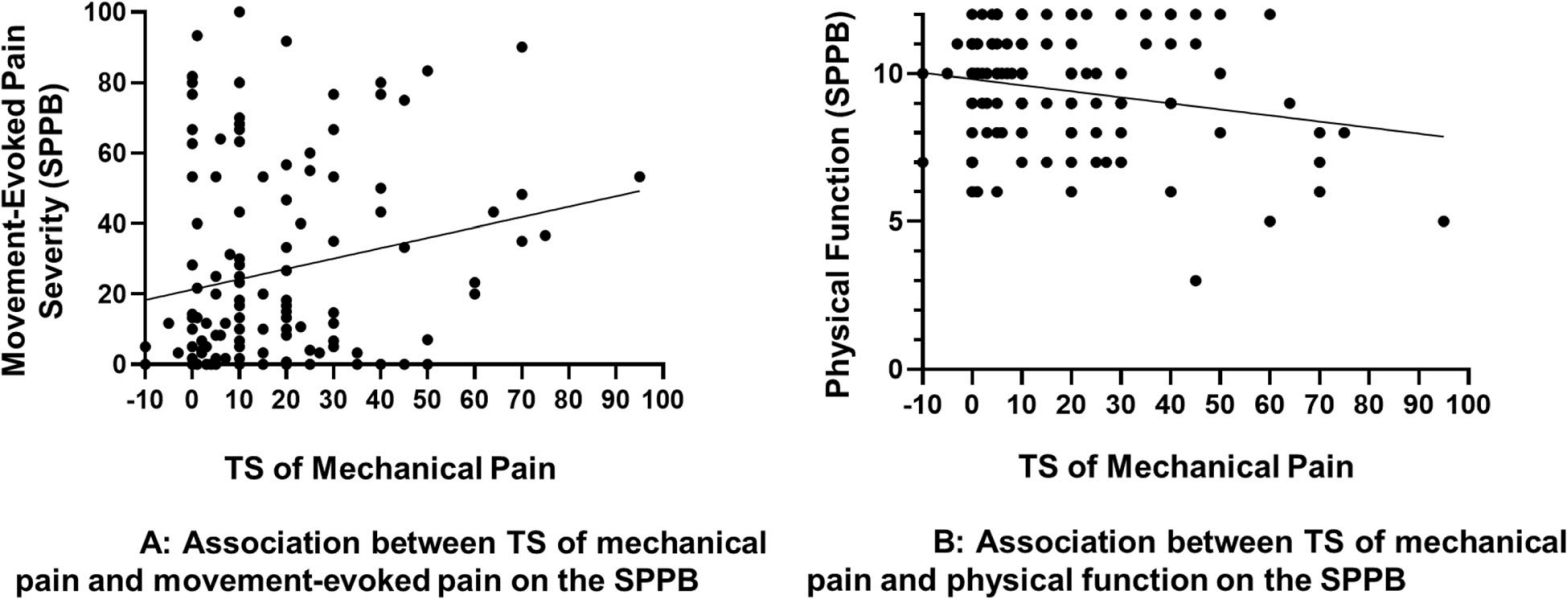
**a** Association between TS of mechanical pain and physical function on the SPPB. **b** Association between TS of mechanical pain and movement-evoked pain on the SPPB

Given the lack of significant correlations among TS, CPM, and self-reported pain severity and interference on the BPI-SF, hierarchical multiple regression models were not analyzed for BPI-SF pain severity and interference. Two hierarchical multiple regression models were analyzed – one for movement-evoked pain and the other for physical function on the SPPB. As presented in Table 4, the overall model predicted approximately 30% of the variance in movement-evoked pain, which was statistically significant (*R*^2^ = .296; F_9,124_ = 5.81, *p* < .001). After adjustment for covariates, greater TS of mechanical pain was found to significantly and prospectively predict greater movement-evoked pain on the SPPB (β = .20, *p* = .016). CPM did not significantly predict movement-evoked pain (β = .02, *p* = .781). These findings suggest that enhanced endogenous pain facilitation may be an important driver of subsequent movement-evoked pain experiences in adults with cLBP (Fig. 2a). Findings revealed that low annual household income (β = −.32, *p* < .001) and greater depressive symptoms (β = .22, *p* = .011) are also likely to be relevant predictors of movement-evoked pain.

**Table 4:** Hierarchical multiple regression predicting movement-evoked pain assessed via the Short Physical Performance Battery

	B	SEB	β	***R***^**2**^	Δ***R***^**2**^	ΔF
**Step 1**				.21	–	8.71**
Sex	−8.03	4.50	−0.15			
Household Income	−2.13	0.55	−0.32**			
Age	0.24	0.16	0.12			
Race	−7.33	4.57	−0.13			
**Step 2**				.26	.05	2.51
BMI	0.24	0.30	0.06			
Current Opioid	−2.82	4.56	−0.05			
Depressive Symptoms	0.58	0.22	0.22**			
**Step 3**				.30	.04	3.47*
TS Mechanical - 512mN	0.28	0.11	0.20*			
CPM	0.02	0.07	0.02			

*BMI* body mass index, *TS* temporal summation, *mN* milliNewton, *CPM* conditioned pain modulation

Sex coded: 1 = Male, 2 = Female

Race coded: 1 = Black, 2 = White

* *p* < 0.05, ** *p* < 0.01.

Results of the second hierarchical regression are presented in Table 5 and show that the overall model significantly predicted approximately 29% of the variance in physical function on the SPPB (*R*^2^ = .285; F_9,124_ = 5.50, *p* < .001). Greater TS of mechanical pain was found to significantly and prospectively predict poorer physical function on the SPPB (β = −.18, *p* = .025) after adjustment for covariates. Similar to the findings for movement-evoked pain, CPM did not significantly predict physical function on the SPPB (β = −.02, *p* = .826). These findings suggest that enhanced endogenous pain facilitation may also contribute to a diminished capacity for physical function in adults with cLBP (Fig. 2b). Results also revealed that poorer physical function on the SPPB was predicted by low annual household income (β = .23, *p* = .007), increasing age (β = −.29, *p* = .001), and greater depressive symptoms (β = −.24, *p* = .006).

**Table 5:** Hierarchical multiple regression predicting physical function assessed via the Short Physical Performance Battery

	B	SEB	β	***R***^**2**^	Δ***R***^**2**^	ΔF
**Step 1**				.18	–	7.18**
Sex	0.05	0.33	0.01			
Household Income	0.11	0.04	0.23**			
Age	−0.04	0.01	−0.29**			
Race	0.51	0.33	0.13			
**Step 2**				.25	.07	3.90*
BMI	−0.04	0.02	−0.15			
Current Opioid	0.19	0.33	0.05			
Depressive Symptoms	−0.04	0.02	−0.24**			
**Step 3**				.29	.04	2.94
TS Mechanical - 512mN	−0.02	0.01	−0.18*			
CPM	−0.00	0.01	−0.02			

*BMI* body mass index, *TS* temporal summation, *mN* milliNewton, *CPM* conditioned pain modulation

Sex coded: 1 = Male, 2 = Female

Race coded: 1 = Black, 2 = White

* *p* < 0.05, ** *p* < 0.01.

## Discussion

The primary objective of this study was to examine whether QST-based tests of endogenous pain modulatory balance might be useful for prospectively predicting future reports of cLBP severity – both movement-evoked pain and self-reported pain on a validated questionnaire. Experimental protocols for the assessment of endogenous pain modulatory balance included TS of mechanical pain and CPM, each assessed at the lumbar spine. TS & CPM protocols were carried out in accordance with commonly recommended methods [19, 30, 37, 38]. Our findings suggest that a pro-nociceptive pain modulatory balance, characterized by a high degree of endogenous pain facilitation (i.e., TS of mechanical pain), may be an important contributor to future episodes of movement-evoked cLBP severity and poorer physical function when assessed with the SPPB. Endogenous pain inhibition (i.e., CPM) did not significantly predict movement-evoked pain severity, which is likely attributable to the overall lack of a significant CPM effect observed in this study. Neither aspect of endogenous pain modulatory balance (TS & CPM) was found to be significantly related to either self-reported pain severity or pain interference on the BPI-SF.

Previous literature has frequently reported high degrees of endogenous pain facilitation (e.g., TS), with concomitant low degrees of pain inhibition (e.g., CPM), as a common characteristic of chronic musculoskeletal pain conditions, like cLBP [39, 40]. Although statistically non-significant, the CPM effect was not completely absent given that some participants with cLBP demonstrated modest CPM effects. Interestingly, greater CPM was significantly correlated with greater TS of mechanical pain in this study. Recall that the CPM effect was not statistically significant in this study; however, this finding suggests that the pain inhibitory processes represented by CPM may have been trying to compensate for an abnormally increased amount of pain facilitation, represented by the statistically significant TS of mechanical pain effect. Over time, it may be that pain inhibitory processes are no longer able to remain in balance with pain facilitatory processes as chronic pain worsens; thus resulting in a pro-nociceptive endogenous pain modulatory balance [41]. Whether the shift to a pro-nociceptive pain modulatory balance is actually antecedent or consequent to chronic pain development is an important topic for understanding the transition from acute to chronic pain. It is not possible for the current study to shed light on this question given that our participants had already developed cLBP. However, it is important to note our study provides evidence that a pro-nociceptive pain modulatory balance, particularly TS of mechanical pain, may predict the perpetuation of cLBP over time. This study nicely compliments previous cross-sectional research correlating TS of mechanical pain to cLBP severity [15].

Findings from this study suggest that endogenous pain facilitation as measured by TS of mechanical pain may be a stronger prospective predictor of movement-evoked pain severity than pain self-reported on a validated questionnaire, at least among individuals with cLBP. It has been suggested that movement-evoked pain and pain reported on a validated questionnaire are not one in the same [21]. This is because, as the name suggests, movement-evoked pain arises upon completion of some physical activity, and its severity is rated in the moment. Most validated questionnaires, like the BPI-SF, ask people to retrospectively recall the severity of their pain while at rest (e.g., rate your worst pain in the last 24 h) [42]. An emerging literature provides evidence of important distinctions between movement-evoked pain and pain at rest, as recalled on validated questionnaires [21]. For example, transcutaneous electric nerve stimulation for individuals with fibromyalgia significantly improved movement-evoked pain but not pain at rest [43]. Peripheral and central sensitization may help explain why TS of mechanical pain was related to movement-evoked pain severity and physical function on the SPPB in this study, but not pain severity or interference reported on the BPI-SF. Peripheral sensitization is an increased sensitivity to an afferent nerve stimuli and plays an important role for increased sensitivity of deep tissue [44]. Central sensitization refers to the phenomenon whereby nociceptive afferents can trigger a prolonged increase in the excitability and synaptic efficacy of neurons in central nociceptive pathways [45]. TS of pain is a widely accepted QST method that has been shown to activate neural mechanisms consistent with peripheral and central sensitization [18, 46]. When peripheral and/or central sensitization is present, generally innocuous movements such as standing from a seated position become sufficient to stimulate nociceptive afferents and produce movement-evoked pain [47, 48], which in turn can compromise physical function. This would help explain why TS of mechanical pain was a significant predictor of movement-evoked pain and physical function on the SPPB in this study. Most validated questionnaires that retrospectively assess pain at rest, and its interference with daily living, do not include a peripheral or central sensitization component. To address this shortcoming, new measures such as the Central Sensitization Inventory have been developed in an attempt to assess various aspects of sensitization via validated questionnaire, especially in studies that are not amenable to inclusion of a QST battery [49, 50]. As it relates to this study, the BPI-SF does not include any specific assessment of peripheral or central sensitization. This may help explain why TS of mechanical pain was not related to pain severity or interference on the BPI-SF.

Consistent with the biopsychosocial model of chronic pain [51], other factors besides endogenous pain modulatory balance were also found to be predictive of movement-evoked pain and physical function on the SPPB. Specifically, low income and greater depressive symptoms were each found to significantly predict greater movement-evoked and poorer physical function, while increasing age also predicted poorer physical function. Evidence suggests that older age [52], limited socioeconomic resources [53], and depressed mood [54] may each heighten risk for poor cLBP outcomes. Further, increasing age [55], poverty [29], and depression [56] may augment peripheral and/or central sensitization, thereby exacerbating movement-evoked pain and physical function limitations. Taken together, older age, poverty, depression, and a pro-nociceptive pain modulatory balance may represent a biopsychosocial phenotype of vulnerability for poor cLBP outcomes; however, additional research is needed to confirm this hypothesis.

As demonstrated in Tables 4 and 5, TS of mechanical pain and CPM together accounted for a modest 4% of the variance in movement-evoked pain and physical function, respectively. Although TS of mechanical pain was a statistically significant predictor of movement-evoked pain and physical function, the modest amount of variance accounted for rightfully calls in to question the clinical relevance of TS of mechanical pain. Importantly, our findings coincide with a growing body of evidence that collectively attests to the clinical relevance of laboratory-based assessments of endogenous pain modulatory balance using TS and CPM protocols. It appears both TS and CPM have value for prospectively predicting chronic pain development as well as the severity of chronic pain over time. For example, greater pre-surgical TS of mechanical pain predicted the development of chronic pain 12 months following total knee arthroplasty in patients with knee osteoarthritis [57]. In patients who underwent thoracotomy, less efficient CPM measured pre-surgically predicted the development of chronic pain at the surgery site approximately 6 months following the procedure [58]. In studies that examined whether endogenous pain modulatory balance prospectively predicts chronic pain severity once it had already developed, it was found that TS of mechanical pain assessed at the knee significantly predicted weekly diary ratings of average clinical pain across 4 weeks in patients with knee osteoarthritis [29]. Our findings add to this body of clinically-relevant literature by showing that TS of mechanical pain assessed at the lumber region of people with cLBP predicts their movement-evoked pain severity and physical function assessed 1-week later.

Despite the clinical relevance described above, this study (and others like it) have practical limitations that need to be addressed. For example, many of the QST protocols for the assessment of TS and CPM require expensive equipment and protocols that are technically complex and time consuming. As such, research involving QST is often carried out in specialized laboratories with highly trained technicians who can operate the equipment. This generally precludes protocols for the assessment of endogenous pain modulatory balance from being widely implemented in the clinical settings where patients present for pain treatments. Recent research has attempted to develop a more clinic-friendly “bedside” QST protocol for use in clinical trials and clinical practice [59]; however, it remains to be determined whether this will be an acceptable approach going forward. Another limitation of this research relates to the current lack of consensus regarding how best to quantify endogenous pain modulatory balance for inclusion in predictive models of future chronic pain outcomes. Endogenous pain modulation represents a complex interplay of top-down and bottom-up inhibitory and facilitatory processes [60]. Yet in the laboratory, researchers tend to measure these processes separately using TS and CPM protocols. Moreover, TS and CPM tend to be examined separately in data analytic models, which arguably does not capture the interactive nature of endogenous pain modulation. Whether novel experimental and/or data analytic methods might be able to better approximate the dynamic interplay of pain inhibitory and facilitatory processes in research addressing endogenous pain modulatory processes is an area in need of greater attention. Furthermore, in this study TS of mechanical pain and CPM were assessed at the sight of maximal pain (i.e., the low back) but not at a distal reference site. Therefore, we cannot differentiate peripheral from central sensitization as a possible explanation linking TS of mechanical pain with movement-evoked pain and physical function on the SPPB. Lastly, our study sample was comprised primarily of African Americans (61.2%), and the largest proportion of the sample (33.8%) fell within the lowest annual household income bracket ($0 - $19,999). Therefore, our study findings may not generalize well to Caucasian populations, or those with higher socioeconomic status (SES). Importantly, African Americans and those with low SES tend to be the most vulnerable to the deleterious effects of chronic pain [61]. Additional cLBP research focused specifically on African Americans and individuals with low SES seems warranted.

## Conclusions

In conclusion, our findings add to the existing body of literature attesting to the clinical relevance of endogenous pain modulatory balance for predicting cLBP outcomes, like movement-evoked pain and physical function. The inclusion of endogenous pain modulatory balance assessment, as well as consideration of sociodemographic (e.g., annual household income, age) and clinical variables (e.g., depressive symptoms), may help improve the overall ability of a clinical assessment to identify people at greatest risk for poor cLBP outcomes [62].

Although beyond the scope of this study, future research should consider investigating the role of sex, gender identity, and ethnicity/race to further understand if, and how, these important individual difference factors affect endogenous pain modulatory balance and its impact on cLBP. Additional research should specifically focus on the time interval between the assessment of endogenous pain modulatory balance and subsequent movement-evoked pain. In the current study it was only 1 week, but additional research could address how long into the future movement-evoked pain can be predicted (e.g., 1 month, 1 year). Lastly, more studies are needed to further elucidate the extent to which specific aspects of endogenous pain modulation interact with other underlying biopsychosocial mechanisms that contribute to poor cLBP outcomes.

## Data Availability

The datasets used and/or analysed during the current study are available from the corresponding author on reasonable request.

## Abbreviations

cLBP: Chronic low back pain
QST: Quantitative sensory testing
TS: Temporal summation
PPT: Pressure pain threshold
CPM: Conditioned pain modulation
SPPB: Short Physical Performance Battery
BPI-SF: Brief Pain Inventory – Short Form
CES-D: Center for Epidemiologic Studies – Depression Scale
BMI: Body mass index
